# Oxidative Stress in Liver Metabolic Dysfunction and Diseases, with a Focus on Hepatogenic Diabetes: Effect of Alcohol Consumption

**DOI:** 10.3390/antiox14121494

**Published:** 2025-12-12

**Authors:** Martha Lucinda Contreras-Zentella, Lorena Carmina Hernández-Espinosa, Rolando Hernández-Muñoz

**Affiliations:** Departamento de Biología Celular y Desarrollo, Instituto de Fisiología Celular, Universidad Nacional Autónoma de México (UNAM), Colonia Copilco, Delegación Coyoacán, Ciudad Universitaria, Ave. Universidad # 3000, Ciudad de México 04510, Mexico; mcontre@ifc.unam.mx (M.L.C.-Z.); lorenah@ifc.unam.mx (L.C.H.-E.)

**Keywords:** liver-pancreas axis, MASLD, insulin resistence, oxidative stress

## Abstract

Metabolic dysfunction–associated fatty liver disease (MASLD) is associated with severe forms of liver injury, including fibrosis and cirrhosis. The main risk factors for MASLD—obesity, type 2 diabetes mellitus (T2DM), dyslipidemia, and insulin resistance (IR)—contribute to metabolic disturbances that initiate hepatic steatosis. Metabolic and alcohol-related liver disease (MetALD) describes patients with MASLD who also present alcohol-associated hepatic injury. Chronic oxidative and inflammatory stress promotes the progression of steatosis in both conditions. T2DM and chronic alcohol consumption are independent lifestyle-related risk factors for cirrhosis within the spectrum of metabolic dysfunction–related liver disease (MASLD and MetALD). The coexistence of both conditions may exacerbate hepatic pathological alterations. IR, which is frequently observed in patients with cirrhosis, can lead to the development of a condition known as hepatogenic diabetes (HD). HD is characterized by hyperinsulinemia, IR, and β-cell dysfunction occurring during the onset of cirrhosis and is associated with hepatic inflammation even in the absence of traditional metabolic risk factors such as obesity or a prior history of T2DM. In this context, alcohol intake enhances lipolysis in peripheral tissues, promotes hepatic steatosis, and aggravates metabolic dysfunction, ultimately contributing to excessive mitochondrial production of reactive oxygen species (ROS). Therefore, the present review examines the role of oxidative stress—both alcohol-related and non-alcohol–related—in the pathogenesis of HD, with particular emphasis on ethanol metabolism, oxidative stress, and their interactions in conditions such as T2DM and MetALD.

## 1. Introduction

Since 2023, international consensus panels have recommended replacing the term non-alcoholic fatty liver disease (NAFLD) with metabolic dysfunction-associated fatty liver disease (MAFLD), a designation that more accurately reflects the metabolic basis of the condition [1,2]. This change in terminology also led to the recognition of a new subgroup within the broader category of steatotic liver disease: metabolic and alcohol-related liver disease (MetALD), the alcohol-associated counterpart of MAFLD. MetALD describes individuals who exhibit hepatic steatosis and metabolic dysfunction and who consume alcohol above established threshold levels, representing an overlap between metabolic and alcohol-induced liver injury [3]. Subsequently, the MASLD Research Center within the Division of Gastroenterology and Hepatology at the University of California San Diego (La Jolla, CA, USA) proposed the term metabolic dysfunction–associated steatotic liver disease (MASLD) as a replacement for metabolic dysfunction–associated fatty liver disease (MAFLD). Both terms describe disorders characterized by alterations in fat metabolism. Several authors have advocated for adopting the term MASLD; therefore, it will be used throughout this review [4].

Ingested alcohol is primarily metabolized in the liver through oxidative pathways that generate acetate, which is subsequently converted into acetyl-CoA. Approximately 90% of systemic alcohol is eliminated by alcohol dehydrogenase (ADH) activity in the gut and liver [5]. Excessive alcohol intake also induces the expression and activity of the microsomal enzyme cytochrome P450 2E1 (CYP2E1), resulting in the production of toxic metabolites capable of promoting oxidative damage to membrane lipids and other macromolecules, including those required for mitochondrial integrity and function [6,7]. In addition, chronic alcohol consumption contributes to systemic oxidative stress, inflammation, acetaldehyde and adduct formation, disruption of the gut barrier, pro-fibrotic tissue remodeling, and mitochondrial dysfunction [8,9].

Alcohol consumption appears to be associated with the risk of developing type 2 diabetes mellitus (T2DM) [10,11]. Despite this recognition, relatively little attention has been given to how alcohol intake affects glycemic control in individuals already diagnosed with T2DM. Previous studies have reported that light to moderate alcohol consumption is associated with a reduced risk of developing T2DM, whereas heavy alcohol intake does not confer such protection [12]. These observations may reflect the potential beneficial effects of ethanol on improving insulin sensitivity and reducing systemic inflammation [11]. Indeed, in patients with autoimmune liver disease, the risk of developing T2DM can be reduced by up to 60% through healthy lifestyle practices, including maintaining a normal body weight, abstaining from smoking, engaging in regular physical activity, adhering to a balanced diet, and consuming alcohol in moderation [13].

The principal mechanisms mediated by alcohol and its metabolites include oxidative stress, inflammation and immune-metabolic deregulation, increased intestinal permeability and dysbiosis, cellular apoptosis and necrosis, extracellular matrix remodeling, endoplasmic reticulum stress, mitochondrial dysfunction, and epigenetic alterations. These mechanisms are highly complex and interdependent, and understanding their interplay is essential for elucidating how they synergistically or additively contribute to alcohol-induced multi-organ injury [14].

Conversely, alcohol consumption is known to promote the release of danger-associated molecular patterns (DAMPs), such as uric acid and extracellular adenosine triphosphate, both of which serve as potent activators of the NLRP3 inflammasome [15]. Despite significant advances in this field, the development of novel therapeutic strategies remains hindered by an incomplete understanding of the regulatory mechanisms governing NLRP3 inflammasome activation [16].

MetALD and MASLD share the common feature of hepatic fat accumulation, which predisposes individuals with simple steatosis (fatty infiltration) to more severe forms of liver injury, including steatohepatitis (fat accumulation accompanied by inflammation), fibrosis, cirrhosis, and hepatocellular carcinoma [17]. In addition, acetaldehyde has been shown to inhibit fatty acid oxidation, thereby promoting triglyceride accumulation within hepatocytes [18]. The primary risk factors for MASLD include obesity, T2DM, dyslipidemia, and insulin resistance (IR), all of which contribute to metabolic disturbances that initiate hepatic steatosis. Chronic oxidative and inflammatory stress associated with fatty liver drives the progression of FLD in both alcoholic and non-alcoholic contexts [19].

Patients with liver cirrhosis often exhibit varying degrees of insulin resistance (IR), which may eventually progress to hepatogenous diabetes (HD) [20]. Liver injury can disrupt glucose metabolism and, in severe cases, lead to the development of diabetes [21,22]. Recent studies have identified hyperinsulinemia as a characteristic clinical feature of cirrhosis [23,24]. When hepatocytes are damaged—particularly in the cirrhotic state—enzymes involved in glucose metabolism display characteristic alterations [25,26].

HD typically presents distinct clinical features: it is frequently associated with hypoglycemic episodes and shows limited correlation with traditional risk factors such as age, body mass index (BMI), or family history of diabetes. Unlike type 2 diabetes mellitus (T2DM), HD arises as a direct consequence of chronic liver disease rather than genetic predisposition. Its pathophysiology involves both β-cell dysfunction and reduced insulin sensitivity. Furthermore, studies have shown that patients with hepatitis B–related cirrhosis exhibit impaired insulin secretion [27].

Therefore, this review aims to examine the role of alcohol consumption in the pathogenesis of HD, with particular emphasis on ethanol metabolism and oxidative stress—conditions in which excess reducing equivalents may exacerbate metabolic deregulation, particularly in the context of T2DM and MASLD.

## 2. Risk Factors for Liver Diseases

### 2.1. Alcohol Consumption

Xenobiotics are chemical substances foreign to living organisms and not naturally produced by the body. Examples include pollutants, pesticides, pharmaceuticals, and chemicals such as ethanol. These compounds can enter the body through ingestion, inhalation, or dermal absorption. Although the body attempts to eliminate them through metabolic detoxification reactions, in some cases their metabolism can increase toxicity. Many xenobiotics are absorbed and metabolized in the intestine and liver [28], processes that can lead to the generation of reactive oxygen species (ROS) [29].

**Figure 1:**
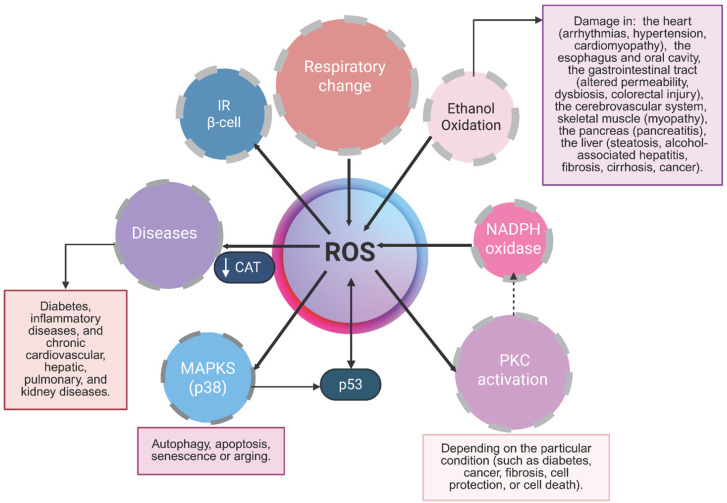
**Signaling pathways activated by reactive oxygen species (ROS) and their roles in pathological conditions.** Mitochondrial ROS are generated through multiple mechanisms, including the activity of Electron Transport Chain Complexes I–III. In the cytoplasm, ROS production primarily depends on the NADPH oxidase (NOX) family, whose members generate superoxide via NADPH-dependent electron transfer. Additionally, the endoplasmic reticulum contributes to ROS formation by producing H_2_O_2_ through the transfer of electrons—mediated by a FAD cofactor—to molecular oxygen. Once formed, ROS activate several intracellular signaling cascades, including protein kinases and mitogen-activated protein kinases (MAPKs). In the context of insulin resistance and diabetes, ROS amplify inflammatory responses by promoting the release of proinflammatory mediators. Ethanol oxidation to acetaldehyde—catalyzed by alcohol dehydrogenase, catalase, or cytochrome P450—constitutes another major source of ROS.

Ethanol is considered a toxic compound whose excessive consumption impairs cognitive function, causes significant health problems, and contributes to millions of deaths worldwide. Its intake has been associated with increased morbidity and mortality across more than 200 diseases [30]. Ethanol remains one of the most widely consumed psychoactive substances globally, used not only by adults but also by adolescents and, in some cases, children. Chronic ethanol consumption can induce damage at multiple systemic and organ levels, including the heart (arrhythmias, hypertension, and cardiomyopathy), the esophagus and oral cavity, the gastrointestinal tract (altered permeability, dysbiosis, and colorectal injury), the cerebrovascular system, skeletal muscle (myopathy), the pancreas (pancreatitis), and the liver (steatosis, alcohol-associated hepatitis, fibrosis, and cirrhosis). In several of these organs, long-term alcohol exposure can also promote carcinogenesis (Figure 1).

MASLD and its alcohol-related counterpart, MetALD, have emerged as leading causes of chronic liver disease worldwide [31]. Excessive alcohol consumption and metabolic dysfunction–associated liver disease represent the most common etiological factors underlying cirrhosis, a major contributor to morbidity and mortality across populations. Cirrhosis typically progresses through two distinct clinical phases: compensated (asymptomatic) and decompensated, depending on the presence or absence of complications such as chronic alcohol abuse or T2DM [32].

### 2.2. Ethanol Metabolism and Oxidative Stress

Blood ethanol concentrations in humans can range from as low as 1 mM, which typically produces no noticeable effects, to levels exceeding 100 mM, which may be potentially lethal. Historically, class I alcohol dehydrogenase (ADH1) has been considered the primary enzyme responsible for ethanol oxidation, whereas other ADH isoenzymes (classes II–V) were thought to play minimal roles [33]. However, the Michaelis constant (Km) of ADH1 for ethanol is relatively low—approximately 1 mM in rodents and 0.05–4.0 mM in humans [34]—and ADH1 has been reported to become inhibited at ethanol concentrations around 20 mM [35]. Consequently, under conditions of chronic or high ethanol intake, ADH1 is unlikely to account for the majority of ethanol metabolism.

Kinetic studies have suggested that ADH III (also known as ADH5) and ADH IV (also known as ADH7), both of which exhibit low affinity for ethanol, may contribute to ethanol oxidation at higher concentrations, such as those occurring during chronic exposure [33]. Nonetheless, other reports indicate that their overall contribution to ethanol metabolism remains limited. Notably, ADH IV appears to preferentially oxidize long-chain alcohols, including retinol, rather than ethanol [36].

Between the late 1970s and early 1990s, new models and experimental findings challenged the long-held view that ADH1 is the dominant ethanol-oxidizing enzyme. While ADH1 efficiently metabolizes ethanol at low concentrations, its activity diminishes at higher or sustained ethanol levels. To delineate the metabolic pathways involved in ethanol oxidation, researchers employed selective enzyme inhibitors. Interestingly, experiments using fomepizole—a specific ADH1 inhibitor—demonstrated residual ethanol oxidation even in the presence of ADH1 inhibition. These findings strongly suggest that alternative enzymatic pathways contribute to ethanol metabolism under conditions of elevated or chronic ethanol exposure [7,35].

It has been postulated that CYP2E1, a member of the cytochrome P450 enzyme family and a key component of the microsomal ethanol oxidizing system (MEOS), along with the catalase pathway, plays a significant role in the metabolism of xenobiotics, including ethanol and free fatty acids (FFAs) [37]. Both pathways are active within microsomes and are upregulated under various pathological conditions, such as diabetes, obesity, starvation, cancer, alcoholic liver disease, and non-alcoholic hepatic steatosis [38,39]. Under conditions of moderate or chronic alcohol consumption, the MEOS pathway—primarily via CYP2E1—contributes to ethanol metabolism by generating acetaldehyde and ROS, particularly the superoxide anion (O_2_^•−^). Upregulation of this pathway has been closely associated with rapidly progressive steatotic hepatitis and subsequent hepatic injury [40].

**Figure 2:**
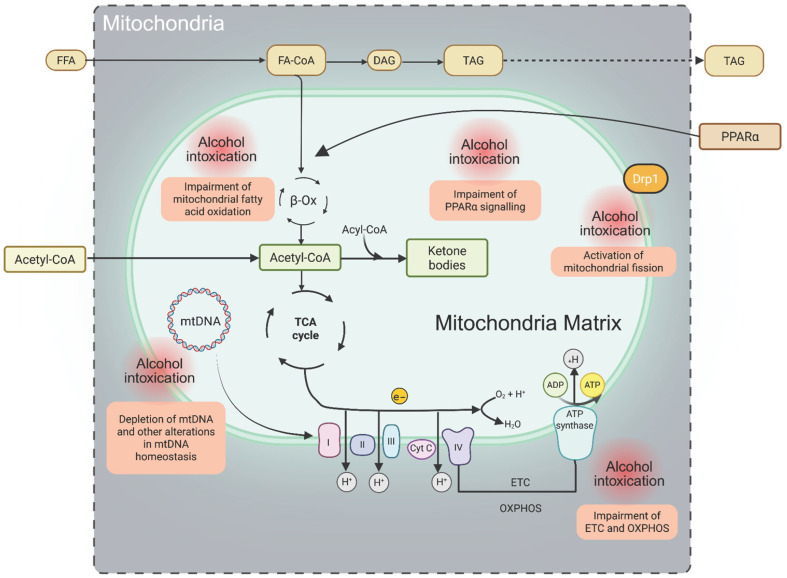
**Key mitochondrial functions and alterations induced by xenobiotics (ethanol).** Long-chain fatty acids (LCFAs) are first activated to fatty acyl-CoA and subsequently transported into mitochondria for β-oxidation through the coordinated action of four enzymes, including NAD- and FAD-dependent dehydrogenases. The resulting electrons are transferred to the electron transport chain (ETC), where they drive proton (H^+^) translocation into the intermembrane space, generating an electrochemical gradient that fuels ATP synthesis via oxidative phosphorylation (OXPHOS). Steps highlighted in red denote mitochondrial metabolic processes particularly vulnerable to disruption by ethanol metabolism. Abbreviations: FFA, free fatty acids; FA-CoA, fatty acyl-CoA; DAG, diacylglycerol; TAG, triacylglycerol; ETC, electron transport chain; OXPHOS, oxidative phosphorylation; mtDNA, mitochondrial DNA; PPARα, peroxisome proliferator-activated receptor-α.

The MEOS pathway is tightly linked to lipid peroxidation processes due to its high ROS production, establishing a functional connection with catalase activity [6,35,41]. Furthermore, studies in rat liver microsomes suggest that long-chain FFAs (C8–C22), which have limited access to mitochondrial β-oxidation, undergo partial oxidation within microsomes to generate shorter-chain FFAs and acetyl-CoA. The resulting acetyl-CoA is subsequently metabolized in mitochondria, where its oxidation may further contribute to ROS generation through respiratory chain activity (Figure 1 and Figure 2).

Another important aspect to highlight is that catalase exhibits activities beyond its classical catalytic function. Under certain conditions, catalase can metabolize low-molecular-weight alcohols via its peroxidatic activity, producing acetaldehyde in the case of ethanol. Increased catalase peroxidatic activity has been reported under conditions of low hydrogen peroxide (H_2_O_2_) generation and elevated ethanol concentrations [42,43], circumstances commonly associated with chronic ethanol consumption [44]. Moreover, the transcriptional promoter of the rat catalase gene contains a peroxisome proliferator–responsive element (PPRE), suggesting that catalase expression can be induced by peroxisome proliferators [45]. Although some studies have indicated that catalase does not serve as the principal ethanol-oxidizing enzyme, when this pathway is active, its catalytic efficiency depends on intracellular H_2_O_2_ concentration [46] (Figure 1).

While ADH1 serves as the primary enzyme for ethanol metabolism under physiological conditions—namely, low ethanol concentrations and the fed state—substantial evidence indicates that under high ethanol exposure or during fasting, alternative pathways such as MEOS (CYP2E1-mediated), catalase, and potentially ADH III become major contributors to ethanol oxidation. Regardless of the metabolic route, ethanol oxidation generates acetaldehyde and reactive oxygen species (ROS). In individuals who consume alcohol, acetaldehyde concentrations in the stomach have been shown to increase up to 6.5-fold.

Beyond its well-documented carcinogenicity, acetaldehyde promotes chronic inflammation and oxidative stress, which in turn induces CYP2E1 expression, further amplifying ROS generation. This cascade contributes to lipid peroxidation, DNA damage, impaired antioxidant defense and DNA repair mechanisms, disrupted methyl transfer processes leading to DNA hypomethylation, reduced hepatic retinoic acid levels, iron overload, and immune system dysfunction. Furthermore, deficiency of aldehyde dehydrogenase-2 (ALDH2) exacerbates the development of alcohol-associated hepatocellular carcinoma.

Hepatocytes contain numerous enzymes essential for lipid, glucose, and protein metabolism, which are central to maintaining energy homeostasis. This homeostasis can be significantly disrupted by the products of ethanol oxidation. Multiple studies indicate that the metabolic pathways engaged in ethanol oxidation depend on several factors, including nutritional status (e.g., the concentration and composition of circulating free fatty acids) and the amount of ethanol ingested [31].

The products of ethanol oxidation—acetaldehyde and reactive oxygen species (ROS)—exert profound effects on cellular metabolism. These molecules contribute to cellular injury through mechanisms such as lipid peroxidation, protein oxidation, and mitochondrial damage. Elevated ROS levels and redox imbalance directly impair mitochondrial proteins and DNA, enhance inflammatory signaling, promote fibrogenesis, and activate apoptotic pathways, thereby intensifying oxidative stress and eliciting compensatory antioxidant responses [47].

Moreover, chronic alcohol exposure in aldehyde dehydrogenase 2 (ALDH2)-deficient hepatocytes leads to the accumulation of oxidized mitochondrial DNA, which is subsequently released via extracellular vesicles. These vesicles can activate multiple oncogenic pathways, thereby promoting hepatocellular carcinoma development [48]. In addition, alcohol disrupts the intestinal epithelial barrier, increasing gut permeability and facilitating the translocation of microbial products and proinflammatory mediators into the liver. This gut–liver axis dysfunction contributes to hepatic inflammation, oxidative stress, and progressive liver injury [49].

### 2.3. Lipid Metabolism

Poly(ADP-ribose) polymerases (PARPs) and tankyrases (TNKS) constitute a family of 17 enzymes with critical roles in cellular homeostasis, DNA repair, and lipid metabolism. Metabolic diseases are frequently associated with inflammation, in which PARP1 activation plays a central role, particularly in MASLD and MetALD. Vascular inflammation also represents a key contributor to the pathogenesis of these disorders [50].

Among PARP family members, PARP1, PARP2, PARP3, TNKS, PARP9, PARP10, and PARP14 have been shown to exert multiple regulatory effects on lipid metabolism. The activity of these enzymes is finely modulated by cholesterol-derived compounds, including oxidized cholesterol derivatives, steroid hormones, and bile acids. PARPs influence several critical processes involved in lipid homeostasis, such as lipotoxicity, fatty acid synthesis, and fatty acid oxidation. Moreover, PARPs act as cofactors for lipid-responsive nuclear receptors and transcription factors, thereby contributing to the regulation of lipid metabolism and energy balance. Dysregulation of PARP activity has been implicated in the pathogenesis of hyperlipidemia, obesity, MASLD, MetALD, and T2DM [51,52].

PARP1 plays a pivotal role in promoting inflammation in both Th1- and Th2-mediated immune responses [52,53]. Its proinflammatory effects have been demonstrated in multiple clinically relevant animal models and corroborated in human studies, highlighting its translational significance [52,53]. PARP1 mediates inflammation through direct and indirect interactions with several proinflammatory transcription factors, most notably nuclear factor κB (NF-κB) [50,54,55].

PARP activation becomes particularly relevant under lipotoxic conditions, such as those observed in MASLD, MetALD, or during toxic hepatic injury (e.g., carbon tetrachloride exposure). Notably, alcohol dehydrogenase (ADH) itself can serve as a target for PARP-mediated modifications [51,56]. A key mechanism underlying lipotoxicity involves oxidative stress–induced DNA damage, which triggers PARP activation [57,58]. Furthermore, de novo lipid biosynthesis has been linked to PARP induction and subsequent activation of extracellular signal–regulated kinases. Consequently, PARP1 is strongly upregulated during high-fat feeding and metabolic overload [51].

Lipotoxicity-induced PARP activation in the liver leads to a decrease in intracellular NAD^+^ levels, which in turn inhibits SIRT1 activity and reduces the expression of liver X receptor-α (LXRα), as well as the activation of the insulin receptor and AMP-activated protein kinase (AMPK)—key regulators of hepatocyte viability and metabolic homeostasis [58,59,60]. In disorders of liver lipid metabolism, PARP activation contributes significantly to lipid accumulation and hepatocellular injury in both MASLD and MetALD. The pathogenic roles of PARP1 and PARP2 are largely analogous in these conditions, with their activation promoting oxidative stress and sustaining hepatic lipid accumulation by suppressing mitochondrial function, particularly fatty acid β-oxidation [51].

In addition to PARP1 and PARP2, other PARP family members such as TNKS and PARP7 (TiPARP) have been implicated in MASLD pathophysiology. TNKS deletion increases the expression of multiple genes involved in lipid uptake and mitochondrial fatty acid oxidation—including *LPL*, *FABP3*, *CD36*, *ACO1*, *CPT1α*, *MCAD*, and *PGC1α*—suggesting enhanced mitochondrial oxidative capacity. Conversely, genetic deletion of PARP7 has been shown to induce hepatic steatosis in mice [61,62].

Pharmacological inhibition of PARPs has been shown to attenuate hepatic inflammation and triglyceride accumulation. In particular, PARP1 inhibition protects against chronic alcohol-induced liver injury by restoring the NAD^+^–SIRT1 signaling axis and preventing the reactivation of sterol regulatory element-binding protein 1 (SREBP1) as well as diacylglycerol acyltransferases 1 and 2 (DGAT1 and DGAT2). Consequently, this intervention suppresses de novo lipogenesis [63,64]. Furthermore, alcohol feeding suppresses peroxisome proliferator-activated receptor alpha (PPARα) expression, an effect that can be partially reversed by PARP inhibition [65,66].

### 2.4. Diabetes

There is a strong association between obesity and T2DM, largely mediated by central nervous system regulation of metabolic pathways. These pathways integrate peripheral and environmental signals to coordinate food intake and energy expenditure, thereby maintaining energy homeostasis [67]. T2DM is a chronic, multifactorial disease influenced by genetic predisposition and environmental factors, and its global prevalence continues to increase. According to the most recent estimates from the NCD Risk Factor Collaboration (2022), approximately 786 million people are currently affected by T2DM worldwide [68].

The pathogenesis of T2DM is primarily characterized by IR and pancreatic β-cell dysfunction, which are further exacerbated by processes such as β-cell dedifferentiation and mitochondrial impairment. Although ROS at physiological levels play an essential regulatory role in insulin gene expression and glucose-stimulated insulin secretion [69], excessive ROS generation induces oxidative stress, contributing to both IR and β-cell damage [70] (Figure 1). The major mechanisms underlying the development of T2DM involve impaired insulin secretion and IR in peripheral tissues [71]. These alterations arise from β-cell dysfunction, which leads to reduced insulin release, deregulated hepatic glucose production, and diminished glucose uptake in skeletal muscle, liver, and adipose tissue. Together, these defects contribute to persistent hyperglycemia [72].

Pancreatic β-cell dysfunction arises from multiple stressors, including endoplasmic reticulum (ER) stress and elevated levels of NADH and ROS associated with chronic hyperglycemia. β-cells are particularly susceptible to oxidative damage due to their high oxygen demand and intrinsically low expression of antioxidant enzymes [73,74]. This vulnerability contributes to glucotoxicity, which accelerates the progression of T2DM by disrupting prostaglandin signaling and promoting mitochondrial dysfunction [75,76].

Consistent with these findings, Fu et al. reported that chronic exposure of pancreatic β-cells to high glucose and palmitate—conditions that replicate glucolipotoxicity—results in impaired insulin secretion and increased ROS production [77,78]. Moreover, mitochondrial abnormalities have been documented in β-cells from patients with T2DM, including smaller, fragmented, and swollen mitochondria, all of which correlate with elevated ROS levels [79,80].

## 3. Metabolic Dysfunction-Associated Steatotic Liver Disease (MASLD) and Metabolic Dysfunction and Alcohol-Associated Steatotic Liver Disease (MetALD)

Hepatic decompensation represents a critical inflection point marking the transition from compensated to decompensated cirrhosis, and its pathophysiological progression differs between alcoholic and non-alcoholic etiologies. Cirrhosis, the final common outcome of chronic and progressive liver fibrosis, accounts for more than one million deaths annually. Alcohol-associated cirrhosis is typically more inflammatory and rapidly progressive, whereas cirrhosis related to metabolic dysfunction–associated fatty liver disease (MASLD) progresses more slowly, driven by insulin resistance, dyslipidemia, and gut-derived inflammatory signals. Persistent alcohol consumption further exacerbates hepatic injury by impairing GAP-induced antimicrobial immunity, an essential intestinal defense mechanism that maintains bacterial compartmentalization within the gut. Disruption of this barrier facilitates bacterial translocation to the liver, thereby worsening alcohol-induced hepatic damage [81,82].

Alcoholic cirrhosis is considered the terminal stage of metabolic dysfunction–associated liver disease with alcohol consumption (MetALD), and follows a pathological sequence that includes steatosis, steatohepatitis, fibrosis, and ultimately cirrhosis. Chronic and excessive alcohol intake (>30 g/day in men and >20 g/day in women) induces hepatocellular injury through multiple converging mechanisms. Toxic intermediates generated during ethanol metabolism, such as acetaldehyde and reactive oxygen species (ROS), promote mitochondrial dysfunction, lipid peroxidation, and DNA damage within hepatocytes. Concurrently, alcohol disrupts intestinal barrier integrity, facilitating the translocation of lipopolysaccharides from the gut microbiota into the portal circulation. This process activates Kupffer cells and sustains chronic hepatic inflammation through toll-like receptor–dependent signaling pathways [83].

MetALD is a chronic hepatic disorder that frequently coexists with other metabolic comorbidities, including alcohol use disorder, type 2 diabetes mellitus (T2DM), hypertension, hyperlipidemia, and obesity. Its development and progression are strongly influenced by lifestyle factors such as poor dietary patterns, physical inactivity, and excessive alcohol consumption [84]. Many chronic liver diseases share common pathogenic mechanisms involving the activation of proinflammatory signaling cascades within the liver. Liver fibrosis, characterized by the excessive accumulation of extracellular matrix proteins, may be driven in part by inflammasome activation [85]. Excessive alcohol intake further exacerbates hepatic steatosis and injury, particularly in individuals with MetALD. One proposed mechanism involves the activation of the NLRP3 inflammasome, as reported by Babuta et al. [86].

Moreover, Blomdahl et al. reported that even moderate alcohol consumption (approximately 30 g/day in men—equivalent to three glasses of wine—and 20 g/day in women—equivalent to two glasses of wine) is associated with advanced fibrosis in patients with MASLD and exerts a synergistic effect with T2DM [87]. Similarly, in MetALD, alcohol consumption has been independently linked to an increased risk of cirrhosis, advanced fibrosis, and higher mortality [88]. MetALD encompasses individuals who meet the diagnostic criteria for steatotic liver disease (SLD) and present both metabolic dysfunction and significant alcohol intake. Specifically, this category includes patients with MASLD who consume alcohol at moderate to high levels, defined as 2–5 standard drinks per day in women and 3–6 standard drinks per day in men [89].

Increased oxidative stress combined with alcohol consumption can further exacerbate hepatic injury, thereby contributing to the development of MetALD [90]. Recent evidence suggests that MetALD may have a poorer prognosis than MASLD alone. Kayaa et al. [89] examined the clinical determinants associated with MetALD severity using data from the National Health and Nutrition Examination Surveys (NHANES 2017–2020), which included 7745 adults to assess the prevalence of MASLD, MetALD, and alcohol-associated liver disease (ALD).

Their analysis revealed that MetALD was present in 4% of participants, whereas MASLD and ALD were identified in 24% and 7%, respectively. The prevalence of fibrosis and advanced fibrosis in MetALD was 10.8% and 3.1%, compared with 24.7% and 9.8% in MASLD. Although MetALD demonstrated the lowest prevalence and fibrosis severity among the three steatotic liver disease subgroups, it may still confer a worse prognosis and higher mortality. This paradox is likely attributable to the heterogeneity in disease progression within this population. Moreover, alcohol consumption and patient age were identified as major determinants of MetALD severity. Higher BMI and the presence of T2DM were also independently associated with an increased risk of fibrosis.

In contrast to these findings, an analysis of a Korean cohort (N = 9497) with MetALD reported an increased risk of developing advanced fibrosis, diverging from the results of Kayaa et al. [89]. Moreover, recent evidence indicates that MetALD has become the third leading indication for liver transplantation in the United States [91,92]. Collectively, these observations suggest that liver disease in individuals with MetALD may progress more rapidly than in those with MASLD, supporting the hypothesis of a synergistic interaction between hepatic steatosis and alcohol consumption. This interaction appears to accelerate fibrosis and increase cancer-related mortality [93].

Therefore, individuals with MetALD who also present T2DM, obesity, or multiple cardiometabolic risk factors are likely to experience a more severe clinical course compared with those who exhibit only a single metabolic abnormality, such as isolated hypertension without accompanying diabetes, obesity, or dyslipidemia. This variability underscores the heterogeneity inherent to MetALD and supports the notion that not all affected individuals follow the same disease trajectory or share a uniform prognosis [89].

The global prevalence of MASLD is estimated to range between 24% and 30%, and this burden continues to rise rapidly in parallel with the worldwide increase in obesity [94]. Metabolic imbalance and IR are common features among individuals with MASLD, and higher BMI together with the presence of T2DM are recognized as major risk factors for the development of advanced fibrosis and steatohepatitis in both MASLD and MetALD [89,95]. The metabolic consequences of these conditions—including oxidative stress driven by ROS, mitochondrial dysfunction, and chronic inflammation associated with hepatic lipid accumulation—contribute to the progression of fatty liver disease. ER stress further promotes inflammatory steatohepatitis by activating Kupffer cells and hepatic stellate cells, which stimulate collagen deposition, ultimately leading to liver fibrosis and, in advanced stages, cirrhosis [96,97].

In older individuals, hepatocytes and non-parenchymal liver cells undergo structural and functional alterations that exacerbate fibrosis, inflammation, steatosis, and metabolic dysfunction, while also impairing detoxification and regenerative capacity. Serum liver enzyme levels often rise due to the cumulative burden of comorbidities; however, paradoxically, declining alanine aminotransferase (ALT) levels have been associated with advanced age, frailty, and increased mortality. Liver diseases are more frequent in the elderly population, with hepatocellular carcinoma and MASLD among the most prevalent. These conditions share pathogenic mechanisms with biological aging, including cardiometabolic alterations such as dyslipidemia, IR, and vascular disease, as well as neurodegenerative processes such as dementia [98] (Figure 1).

## 4. Is Diabetes Related to Alcohol Consumption and Liver Damage?

It is well established that the risk of cirrhosis increases with exposure to xenobiotics such as drugs or ethanol. Recent studies in female Wistar rats have shown that the coexistence of T2DM and chronic alcohol consumption exerts a significant synergistic effect on fatty liver disease (FLD), leading to exacerbated hepatic injury and accelerated progression to cirrhosis [99,100]. In chronic alcohol-associated liver disease (ALD), ethanol oxidation through multiple metabolic pathways generates acetaldehyde, which is subse9uently metabolized to acetyl-CoA. When produced in excess, acetyl-CoA promotes the synthesis of long-chain free fatty acids (FFAs) and increases triglyceride accumulation within hepatocytes [18]. In contrast, the primary drivers of MASLD—obesity, T2DM, dyslipidemia, and IR—induce metabolic disturbances and lipid dysregulation that initiate hepatic steatosis.

Furthermore, T2DM is a well-recognized chronic condition that increases susceptibility to chronic liver disease. Cirrhosis, in turn, disrupts glucose metabolism through multiple mechanisms, while diabetes heightens vulnerability to severe hepatic injury [96]. In a study by Veena et al., the combined effects of alcoholism and diabetes in pre-diabetic and diabetic rats were shown to exacerbate the progression of fatty liver. The alcoholic–diabetic group exhibited significant increases in serum markers of hepatic injury, including aspartate aminotransferase and alkaline phosphatase, compared with controls [17]. In addition, the activities of key antioxidant defense enzymes—such as glutamate dehydrogenase, catalase, superoxide dismutase, and glutathione S-transferase (GST)—were significantly reduced in diabetic animals, with the most pronounced decreases observed in alcoholic–diabetic rats.

This decline in antioxidant capacity was accompanied by increased oxidative stress, as indicated by elevated levels of thiobarbituric acid–reactive substances (TBARS). Moreover, significantly higher hepatic hydroxyproline levels were observed in alcoholic–diabetic animals, reflecting enhanced collagen accumulation and fibrosis. Collectively, these findings demonstrate that alcohol consumption exacerbates fatty liver disease and fibrosis under diabetic conditions. Importantly, the marked reduction in GST activity—an enzyme responsible for conjugating carbonyl compounds involved in the detoxification of lipid-derived aldehydes—suggests impaired neutralization of these reactive molecules, which are both proinflammatory and potentially carcinogenic [101].

In this context, Kikuchi et al. reported that T2DM is a significant risk factor in patients with alcoholic cirrhosis, suggesting that diabetes may aggravate alcohol-associated liver disease through synergistic interactions with chronic alcohol intake [102]. Similarly, Wai et al. found that the prevalence of T2DM was 27% higher among individuals with alcoholic cirrhosis, further implicating metabolic dysfunction in the progression of alcohol-induced hepatic injury. Moreover, alcohol-induced oxidative stress has been linked to increased DNA damage, contributing to hepatocellular injury and fibrosis [103].

Alcohol consumption compromises gut barrier integrity, increasing intestinal permeability and facilitating the translocation of gut-derived proinflammatory mediators to the liver, thereby amplifying hepatic inflammation and injury. Metabolic insults—particularly excessive alcohol intake or diets high in fat and low in dietary fiber—further disrupt intestinal homeostasis in MASLD by altering the gut microbiota and promoting the expansion of potentially pathogenic bacterial species. These microbes induce hepatic immune activation and stimulate hepatic stellate cell activity, contributing to the development of steatohepatitis and T2DM through the portal delivery of pathogen-associated molecular patterns (PAMPs), including lipopolysaccharides, peptidoglycans, and flagellin.

Additionally, dysbiosis enhances intestinal bile acid deconjugation, leading to increased production of secondary bile acids that suppress Farnesoid X Receptor (FXR) signaling [49]. FXR inhibition disrupts bile acid metabolism and lipid homeostasis, further exacerbating hepatic inflammation, fibrosis, and metabolic deregulation.

### Metabolic Affectation Associated with Oxidative Stress in Alcoholism/T2DM

The metabolic consequences of alcohol consumption in patients with T2DM have been widely debated. Acute effects include hypoglycemia, metabolic deregulation, and acidosis, whereas chronic effects encompass hypertension, weight gain, and neuropathy. Some of these alterations arise directly from alcohol or its metabolites, acetaldehyde and acetate, while others stem from alcohol-induced shifts in the hepatocellular NADH/NAD^+^ ratio, which profoundly alter the cellular redox state. This redox imbalance inhibits the citric acid cycle and the β-oxidation of fatty acids, while favoring the conversion of pyruvate to lactate, collectively suppressing hepatic gluconeogenesis [104]. Notably, after the ingestion of approximately 48 g of alcohol (roughly four standard drinks), hepatic gluconeogenesis may decrease by up to 45% [105].

Moreover, studies in patients with diagnosed T2DM who consume ethanol report that, at earlier stages, alcohol intake may reduce or delay the onset of T2DM. The effects of ethanol on carbohydrate metabolism are complex. Diabetic patients may benefit from moderate alcohol consumption due to its influence on lipid metabolism, hemostatic balance, blood pressure, and insulin sensitivity. Ethanol affects glucose metabolism in multiple ways in both diabetic and non-diabetic individuals. Because ethanol inhibits both gluconeogenesis and glycogenolysis, excessive intake during fasting can lead to hypoglycemia, particularly in individuals with depleted glycogen stores. Conversely, when ethanol is consumed with food, the accompanying carbohydrates can increase blood glucose levels and stimulate an insulin response in patients with T2DM.

In cases of moderate alcohol consumption, the risks of disturbances in glycemic control, body weight, and blood pressure are generally limited. However, excessive ethanol intake can disrupt metabolic homeostasis and negate any potential cardiovascular benefits. Epidemiological studies have underscored the complex relationship between moderate alcohol intake and diabetes risk [106]. In addition to hepatic steatosis, patients with MASLD frequently present with one or more cardiometabolic risk factors, including elevated BMI, hypertension, T2DM, dyslipidemia, or hypertriglyceridemia [107,108]. Individuals with diabetes are also more likely to develop hepatic steatosis, a condition associated with an increased risk of liver cancer through mechanisms involving chronic inflammation, oxidative stress, and metabolic deregulation. Hepatic steatosis may further progress to fibrosis, cirrhosis, and ultimately hepatocellular carcinoma [106,109,110].

Elevated serum glucose, hyperinsulinemia, and T2DM are also associated with an increased risk of liver cancer mortality. Insulin may exert mitogenic effects by activating the insulin receptor and downstream intracellular signaling cascades, including the phosphatidylinositol 3-kinase (PI3K)–AKT pathway, which promotes cell proliferation and inhibits apoptosis. Moreover, hyperinsulinemia can elevate circulating levels of free insulin-like growth factor 1 (IGF-1) by reducing hepatic production of IGF-1 binding proteins 1 and 2, thereby further enhancing tumor growth and proliferation [106].

The development of liver disease and hepatic insulin resistance is closely linked to the interplay among free fatty acid accumulation, hepatic inflammation, and increased oxidative stress. In patients with diabetes, hyperglycemia promotes oxidative stress through excessive glucose oxidation and other metabolic pathways, leading to the generation of ROS, including hydroxyl radicals [111]. These ROS can damage DNA, induce gene mutations, and contribute to carcinogenesis. Obesity associated with T2DM further amplifies this process by increasing the production of proinflammatory mediators such as tumor necrosis factor-alpha (TNF-α) and interleukin-6 (IL-6), while reducing adiponectin levels, a hormone with anti-inflammatory and insulin-sensitizing properties. This chronic proinflammatory state accelerates liver injury [112]. In response to oxidative stress and cellular damage, hepatocytes and immune cells release additional proinflammatory cytokines, including interleukin-1β and TNF-α, which initiate and amplify inflammatory signaling, recruit immune cells to sites of injury, and further exacerbate hepatic damage [113].

## 5. Hepatogenic Diabetes

As previously discussed, chronic liver disease is closely associated with diabetes mellitus—particularly T2DM—which substantially increases the risk of developing hepatocellular carcinoma [114]. Naunyn first described the relationship between liver disease and diabetes as hepatogenic diabetes in 1898 [115]. T2DM is characterized by a progressive decline in pancreatic β-cell insulin secretion and increased peripheral insulin resistance, and it is strongly associated with MASLD as a major contributor to chronic liver injury. Although rare, type 1 diabetes—resulting from autoimmune β-cell destruction and complete loss of insulin production—can also contribute to autoimmune hepatitis [116].

The liver plays a central role in glucose homeostasis, and patients with hepatic dysfunction frequently develop impaired glucose tolerance, which may progress to overt diabetes. Liver diseases such as MASLD and MetALD commonly involve chronic inflammation and pancreatic β-cell injury, both of which are strongly linked to T2DM and are associated with worse hepatic outcomes [117,118].

Adipose tissue dysfunction—particularly within visceral fat—along with impaired glucose metabolism and gut microbiota dysbiosis, further exacerbates hepatic injury and insulin resistance. Hepatic lipid accumulation, oxidative stress, and ER stress amplify inflammation and fibrosis, contributing to the severity and progression of liver disease [119]. Since the first description of the association between liver disease and diabetes as hepatogenic diabetes [120], the term hepatogenic or hepatogenous diabetes has been used to describe diabetes that develops after the onset of liver cirrhosis [115].

HD represents a distinct form of diabetes in which liver disease, typically cirrhosis, is often accompanied by portal hypertension and portocaval shunting—which serves as the primary driver of hyperglycemia. The development of diabetes is therefore viewed as a complication arising from cirrhosis. In the setting of liver injury, hyperinsulinemia is influenced by elevated levels of glucagon, growth hormone, insulin-like growth factor 1 (IGF-1), free fatty acids (FFAs), and proinflammatory cytokines [121].

Liver steatosis alters the secretion of multiple cytokines, collectively termed hepatokines, many of which exhibit diabetogenic properties. Key hepatokines include fetuin-A [122], fetuin-B, selenoprotein-P [123], dipeptidyl peptidase-4 (DPP4) [124], and hepatocyte-derived fibrinogen-related protein-1 [125]. These molecules modulate metabolic pathways in the liver, skeletal muscle, adipose tissue, and pancreas, thereby promoting insulin resistance. Fetuin-A, secreted predominantly by the liver, has been implicated in the development of insulin resistance in animal models and is associated with fatty liver in humans, acting as a potential mediator of metabolic dysfunction.

The principal sources of fatty acids include plasma free fatty acids (primarily released from adipose tissue), de novo lipogenesis, and dietary intake [126]. Systemic hypoxia is commonly observed in patients with advanced cirrhosis, because of liver fibrosis, increased intrahepatic vascular resistance, and portal hypertension [127]. Hypoxia has also been linked to the progression of fatty liver disease by inhibiting fatty acid oxidation and promoting lipogenesis [128]. These effects are largely mediated through hypoxia-inducible factors (HIFs), which play a central role in the pathophysiology of chronic liver disease. Excessive HIF activation can exert deleterious effects on pancreatic β-cells, thereby exacerbating metabolic dysfunction [129].

Visceral white adipose tissue (WAT) plays a central role in disrupting glucose homeostasis in MASLD. WAT secretes several hormones and adipokines, including leptin, adiponectin, resistin, TNF-α, and interleukin-6 [130,131,132,133]. Consequently, central obesity—a hallmark of MASLD—contributes to chronic low-grade inflammation that promotes macrophage recruitment, with these immune cells being transported directly to the liver through the portal circulation [134]. This inflammatory milieu is further aggravated by hyperglycemia, hyperinsulinemia, and adipose tissue dysfunction, leading to insulin resistance in peripheral tissues such as muscle, liver, and adipose tissue, as well as pancreatic β-cell impairment. These pathophysiological alterations reduce glucose tolerance and ultimately contribute to the development or worsening of T2DM, progressing in parallel with the severity of liver disease [135].

In addition to the development of HD, traditional risk factors influencing carbohydrate metabolism also play an important role. However, pathogenetic mechanisms inherent to liver disease appear to be particularly relevant in the onset of HD. Despite ongoing research, the precise mechanisms underlying hepatogenic diabetes remain unclear. HD exhibits distinct pathophysiological and clinical features compared with T2DM. Unlike T2DM, HD typically develops in the absence of obesity or other components of the metabolic syndrome, such as dyslipidemia or hypertension. Nevertheless, HD is not considered an independent disease entity [120,121]. A key factor distinguishing HD from T2DM is the temporal relationship between the onset of diabetes and the progression of liver disease. The development of diabetes after the establishment of cirrhosis generally indicates HD, whereas in T2DM, hyperglycemia typically precedes the development of cirrhosis by many years [136].

Several studies have proposed that the primary pathogenic mechanism underlying HD involves the detrimental effects of liver damage on pancreatic islet function. This hypothesis is supported by clinical evidence showing that HD often resolves following successful liver transplantation, indicating that the condition is closely linked to impaired hepatic function rather than intrinsic pancreatic β-cell failure [121]. In HD, mortality is typically driven by liver-related complications, whereas patients with T2DM who subsequently develop chronic liver disease most commonly die from cardiovascular events [137].

Furthermore, the incidence of chronic pancreatitis is strongly associated with excessive alcohol consumption—which accounts for approximately 40–90% of cases—and is also influenced by specific dietary habits. Notably, around 80% of patients with liver cirrhosis exhibit impaired glucose tolerance, and 15–20% ultimately progress to overt diabetes. Thus, it is highly likely that a combination of metabolic, toxic, and secretory disturbances contributes to the development of chronic alcoholic pancreatitis, whereas during acute episodes, the pathogenic mechanisms appear to resemble those observed in acute pancreatitis [138].

## 6. Oxidative Stress and Mitochondrial Damage

Mitochondrial oxidation of metabolic substrates generates NADH and FADH_2_, whose electrons and protons traverse the electron transport chain to establish an electrochemical gradient required for ATP synthesis. This process also regenerates NAD^+^ and FAD, which are essential for sustaining subsequent cycles of substrate oxidation [139]. The primary physiological role of hepatic mitochondria is energy production through the oxidation of substrates such as amino acids, pyruvate, and fatty acids. The coupling between substrate oxidation and ATP generation via oxidative phosphorylation (OXPHOS) is tightly regulated by numerous circulating and intrahepatocellular factors [140,141].

The efficiency of this coupling is commonly assessed by the respiratory control ratio, defined as the ratio of ADP-stimulated (coupled) respiration to basal (resting) respiration, which serves as an indicator of mitochondrial integrity and bioenergetic performance. In adipose tissue, mitochondrial dysfunction contributes to metabolic impairment, characterized by reduced insulin-mediated triglyceride storage. This dysfunction promotes lipid spillover into non-adipose tissues, thereby aggravating systemic metabolic disturbances and IR [142].

Fatty acids are oxidized via the β-oxidation pathway, a process that requires coenzyme A (CoA), L-carnitine, and several chain-length–specific enzymes [143]. During fasting, hepatic mitochondrial fatty acid oxidation generates ketone bodies, which serve as alternative energy substrates and are subsequently oxidized in extrahepatic tissues through the tricarboxylic acid cycle to produce ATP [144]. Mitochondrial homeostasis is additionally regulated by mechanisms that control mitochondrial number and quality, including mitochondrial dynamics (fusion and fission) and mitophagy—a selective type of autophagy responsible for the removal of damaged mitochondria [145]. These regulatory pathways underscore how hepatic lipid accumulation induces dynamic alterations in mitochondrial function, ultimately facilitating the progression of MASLD toward hepatic fibrosis and cirrhosis [146] (Figure 2).

Mitochondria play a central role in cellular signaling through the generation of ROS, produced mainly by electron transport chain complexes I and III, as well as by enzymes involved in mitochondrial fatty acid oxidation [147]. These ROS function as signaling molecules that regulate multiple nuclear transcription factors, including nuclear factor erythroid 2–like 2 (NFE2L2 or Nrf2), a key mediator of antioxidant responses and mitochondrial biogenesis [147,148]. However, in hepatic steatosis, disturbances in mitochondrial function—characterized by impaired fatty acid β-oxidation, excessive lipid influx from extrahepatic tissues, and disrupted mitochondrial dynamics—lead to increased ROS production and dysregulation of lipid metabolism–associated transcription factors. Collectively, these alterations contribute to mitochondrial dysfunction and oxidative stress, which are central drivers of liver injury and progression toward more advanced stages of metabolic-associated fatty liver disease [149,150].

The oxidative capacity of mitochondria varies considerably across the spectrum of obesity and MASLD, influenced primarily by body mass but also by age, insulin sensitivity, coexisting T2DM, chronic alcohol consumption, and potentially genetic variants. Interestingly, mitochondrial oxidative capacity may transiently increase during prolonged obesity, enhancing fatty acid oxidation and thereby limiting triglyceride accumulation [151]. However, this compensatory response concurrently increases oxidative stress, disrupting the balance between oxidative and antioxidative systems. Over time, depletion of antioxidant defenses leads to progressive mitochondrial dysfunction, a hallmark of steatohepatitis and fibrosis. Chronic oxidative stress further activates intracellular inflammatory pathways, promoting both intrahepatic and systemic inflammation [151]. Clinical studies in patients with T2DM have shown that progressive IR is associated with increased mitochondrial fragmentation and reduced oxidative capacity [152]. One of the earliest cellular responses to oxidative stress is the activation of mitophagy, a specialized autophagic process that selectively removes aged, damaged, or dysfunctional mitochondria to preserve mitochondrial quality control [153].

Fatty liver diseases can also be triggered by various xenobiotics, including ethanol, pharmaceuticals, and environmental toxicants. The alterations in mitochondrial dynamics induced by these agents have been most extensively studied in the context of alcohol-related fatty liver disease. Recent experimental evidence indicates that ethanol-induced steatosis may result from aberrant activation of mitochondrial fission through increased expression of dynamin-related protein 1 (Drp1) (Figure 2). Notably, ethanol-stimulated Drp1 expression appears to be driven by upregulation of the orphan nuclear receptor NR4A1 and activation of the p53 signaling pathway [154]. Xenobiotics impair mitochondrial fatty acid β-oxidation through multiple, often concurrent mechanisms, including direct inhibition of enzymes involved in fatty acid oxidation, downregulation of peroxisome proliferator-activated receptor alpha (PPARα) expression and activity, and mitochondrial DNA (mtDNA) damage. Injury to mtDNA compromises electron transport chain function, thereby reducing oxidative phosphorylation efficiency and limiting fatty acid oxidation. These disruptions collectively diminish the availability of NAD^+^ and FAD, further impairing oxidative metabolism and promoting hepatic lipid accumulation [147,155,156,157] (Figure 2).

Mitochondrial dysfunction and oxidative stress may account for the combined deleterious effects of alcohol intake and metabolic dysfunction, primarily through the induction of inflammatory cytokines and activation of the innate immune response [149,150]. Elevated ROS and the resulting oxidative stress act as signals that promote the release of proinflammatory cytokines, including interleukin-1β and TNF-α. These mediators initiate and amplify inflammatory cascades that further damage mitochondrial components such as proteins, lipids, and DNA [112]. Notably, alcohol-related microvesicular steatosis has been strongly associated with these mechanisms [158,159].

Ethanol-induced mitochondrial ROS overproduction may arise not only from reduced efficiency of the electron transport chain but also from increased mitochondrial expression of CYP2E1 [160]. Abnormal mitochondrial function is now widely recognized as a major contributor to xenobiotic-induced exacerbation of MASLD. Experimental studies have shown that obese, ethanol-intoxicated mice exhibit reduced hepatic ATP levels and lower mRNA expression of Nrf1, Ppargc1a (encoding PGC-1α), and PPARA (encoding PPARα). Likewise, in HepaRG cells, the transition from steatosis to a MASH-like phenotype is characterized by excessive mitochondrial ROS generation, decreased mtDNA content, and impaired mitochondrial respiration [151].

### Mitochondrial Dynamics

**Figure 3:**
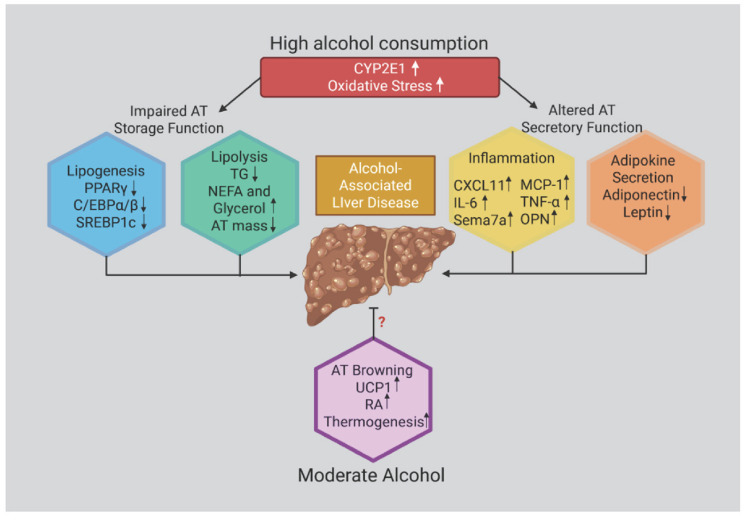
**Effects of ethanol in promoting adipose tissue (AT) dysfunction.** Chronic and heavy ethanol exposure induces oxidative stress, primarily through CYP2E1 activation, which disrupts ATP homeostasis and impairs adipose tissue (AT) secretory functions. Ethanol alters AT endocrine activity by increasing the production and release of proinflammatory mediators—including MCP-1, interleukin-6 (IL-6), tumor necrosis factor-α (TNF-α), and osteopontin—while concurrently dysregulating adipokine secretion. Collectively, these alterations contribute to the onset and progression of Alcohol-Associated Liver Disease (AALD).

Mitochondrial morphology dynamically adapts to cellular metabolic demands through tightly regulated fission and fusion processes, which are governed by post-translational modifications of dynamin-related GTPases. Fusion requires the merging of the outer mitochondrial membranes mediated by mitofusins (MFN1 and MFN2), and the fusion of the inner membrane coordinated by optic atrophy 1 (OPA1). These fusion events enable the exchange of mitochondrial matrix components and stabilize cristae architecture. In addition to complete fusion, transient “kiss-and-run” interactions permit selective inter-mitochondrial communication without full membrane merging. Conversely, mitochondrial fission—driven by the recruitment of dynamin-related protein 1 (Drp1) to mitochondrial surface receptors such as Mff, Mid49/51, and Fis1—facilitates the segregation of damaged or dysfunctional mitochondrial regions, which are subsequently targeted for degradation. Dysregulation of these dynamics—particularly excessive fission or impaired fusion—reduces metabolic flexibility, promotes mitochondrial dysfunction, and exacerbates oxidative stress. To preserve mitochondrial integrity and bioenergetic function, cells rely on additional quality-control pathways, including mitochondrial motility, the formation of mitochondria-derived vesicles, the ubiquitin–proteasome system, and extracellular or environmental signaling cues that fine-tune mitochondrial turnover and remodeling [139] (Figure 2 and Figure 3).

Mitochondrial dynamics involve a coordinated cycle of fission—the division of a single mitochondrion into two daughter organelles—and fusion, the merging of two mitochondria into a continuous network. Excessive mitochondrial fission, which leads to organelle fragmentation, is commonly associated with mitochondrial dysfunction, particularly under conditions of cellular stress or during apoptosis [161]. In diabetes, impaired regulation of mitochondrial dynamics accelerates systemic metabolic deterioration. Key pathogenic features include reduced mitochondrial biogenesis and fusion, excessive fission, and defective mitophagy, all of which compromise mitochondrial integrity and diminish metabolic flexibility [139].

As a key mitochondrial quality-control mechanism, mitophagy selectively identifies and eliminates damaged mitochondria, thereby preventing the accumulation of dysfunctional organelles and limiting excessive mitochondrial ROS production. In doing so, mitophagy preserves mitochondrial function, supports fatty acid β-oxidation, and prevents hepatic lipid accumulation [139,154]. Conversely, impaired mitophagy exacerbates insulin resistance by allowing dysfunctional mitochondria to persist and by activating the NLRP3 inflammasome. Several proteins involved in mitochondrial dynamics exert direct effects on insulin signaling. For example, mitofusin-2 (Mfn2) stabilizes insulin receptor substrate 1 (IRS-1) and facilitates communication between mitochondria and the endoplasmic reticulum, thereby supporting metabolic homeostasis. Likewise, phosphorylation of the mitochondrial fission factor (Mff) enhances hepatic insulin sensitivity by modulating mitochondrial fragmentation. Reciprocally, insulin regulates mitochondrial quality control through the Akt/mTOR/FOXO1 pathway, which suppresses Drp1/Fis1-mediated fission while promoting PGC-1α–driven mitochondrial biogenesis.

Disruption of this bidirectional regulatory network ultimately contributes to the progression of metabolic disease. For instance, mitophagy-deficient pancreatic β-cells display impaired insulin secretion, illustrating how deficits in mitochondrial quality control generate a vicious cycle involving insulin resistance, oxidative stress, and progressive mitochondrial dysfunction [139].

Mitochondrial dynamic deregulation under conditions of metabolic stress, chronic hyperglycemia, and lipid overload impairs oxidative phosphorylation, leading to elevated ROS production and reduced ATP synthesis. Excessive ROS induces serine phosphorylation of insulin receptor substrates 1 and 2 (IRS1/2), thereby disrupting insulin signaling and promoting both β-cell apoptosis and peripheral insulin resistance. In T2DM, downregulation of PGC-1α and NRF1 further diminishes mitochondrial biogenesis, fatty acid oxidation, and respiratory capacity. At the same time, hyperglycemia-driven upregulation of fission proteins accelerates mitochondrial fragmentation, exacerbating oxidative stress and perpetuating mitochondrial dysfunction [139,162] (Figure 2 and Figure 3).

## 7. Conclusions

Alcohol is primarily metabolized in the liver through oxidative pathways to produce acetate, which is subsequently converted to acetyl-CoA. However, excessive alcohol consumption induces the expression and activity of microsomal CYP2E1, promoting the formation of toxic metabolites and ROS. Alcohol intake further contributes to cellular oxidative stress, inflammation, acetaldehyde adduct formation, gut barrier disruption, profibrotic tissue remodeling, and systemic mitochondrial dysfunction. Epidemiological evidence indicates a complex association between alcohol consumption and T2DM risk: while light to moderate intake appears to exert a protective effect, chronic or excessive consumption leads to metabolic deregulation and hepatic injury. Both alcoholic and non-alcoholic fatty liver diseases (ALD and NAFLD) share the hallmark feature of hepatic lipid accumulation, which predisposes individuals to progressive liver injury, ranging from simple steatosis to steatohepatitis, fibrosis, and ultimately cirrhosis. In contrast, obesity, T2DM, dyslipidemia, and insulin resistance, all of which promote hepatic lipid deposition and metabolic dysfunction, predominantly drive NAFLD.

**Figure 4:**
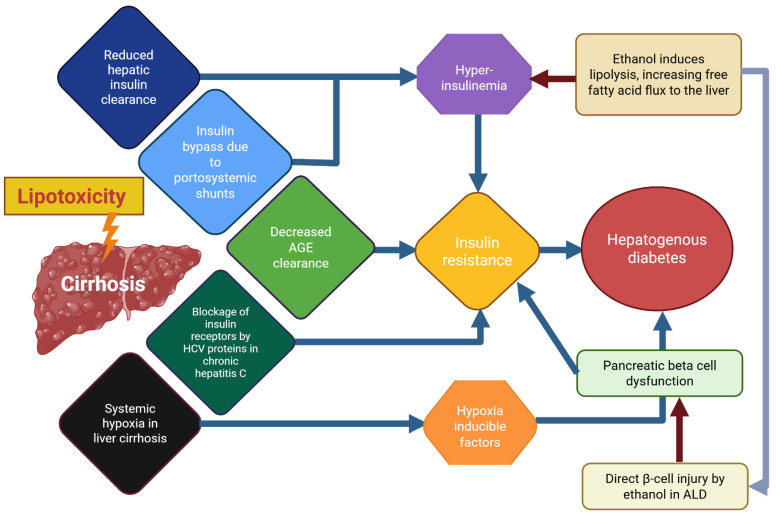
**A scheme or “graphical abstract” of probable mechanism underlying hepatogenic diabetes and the involvement of ethanol effects in this pathological entity.** This scheme provides a simplified overview of the pathogenesis of hepatogenic diabetes, highlighting the central roles of insulin resistance and β-cell dysfunction in disease development. It also illustrates the multiple factors that impair liver function and collectively contribute to metabolic deregulation. For further details, refer to the main text and the legends of Figure 1 and Figure 3. Abbreviations: AGEs, advanced glycation end products; ALD, alcohol-related liver disease; HCV, hepatitis C virus; NAFLD, non-alcoholic fatty liver disease.

Patients with liver cirrhosis frequently develop varying degrees of IR, which may progress to hepatogenic diabetes (HD) (Figure 4). Notably, HD displays distinct clinical features and is often accompanied by hypoglycemic episodes resulting from impaired hepatic glucose regulation in chronic liver disease. Mitochondrial oxidative capacity plays a central role in the onset and progression of obesity and NAFLD and is influenced by factors such as body mass, age, insulin sensitivity, coexisting T2DM, chronic alcohol intake, and possibly genetic variants. These factors collectively enhance oxidative stress, disrupting the balance between oxidant and antioxidant systems.

Overall, alterations in lipid and carbohydrate metabolism, pancreatic β-cell dysfunction, impaired intestinal permeability, and alcohol consumption with subsequent oxidative metabolism disrupt cellular homeostasis through multiple mechanisms. Despite their distinct origins, these alterations converge on a common outcome: IR, which can ultimately lead to T2DM. Protein and DNA damage, mitochondrial dysfunction, and immune deregulation further promote fibrogenesis and activate apoptotic pathways. Consequently, cirrhosis associated with MASLD—and alcoholic cirrhosis, the terminal stage of MetALD—progresses in parallel, with both strongly linked to increased ROS production and inflammation. This is particularly relevant in HD, where many of these pathogenic factors coexist, although their mechanistic contributions may differ. Therefore, further research is warranted (Figure 4).

## 8. Recommendations for Future Research

The present review aimed to update current knowledge on HD, a clinically relevant condition in which alcohol-induced liver injury may play an important role. The liver and pancreas are interconnected through the liver–pancreas axis, which is essential for glucose regulation. HD develops when liver disease—particularly cirrhosis—disrupts this axis, leading to insulin resistance and pancreatic β-cell dysfunction, ultimately causing hyperglycemia (Figure 3). This condition represents a form of diabetes that arises because of liver failure and is distinct from both type 1 and type 2 diabetes. Notably, its prevalence increases in parallel with the severity of liver disease.

In this review, we attempted to synthesize current evidence to elucidate the mechanisms linking alcohol-induced liver injury to HD (Figure 3). However, our analysis indicates that the most clearly established mechanisms continue to involve ethanol metabolism, oxidative stress, and liver injury. Thus, the present work may serve as a foundation to highlight mechanisms that require further investigation. Among the most relevant topics for future research is the temporal sequence of pathogenic events—specifically, whether chronic alcohol-induced liver damage precedes pancreatic injury that leads to insulin resistance, or whether a persistent inflammatory state, together with ethanol metabolism, promotes insulin resistance and subsequently contributes to fatty liver disease.

Patterns of alcohol consumption among individuals with HD remain poorly defined, and little is known regarding the pharmacokinetics and pharmacodynamics of ethanol in diabetic patients. Critical unanswered questions include whether ethanol oxidation itself is the primary driver of diabetes, or whether its reactive metabolites play a more dominant role, as well as the extent to which acetaldehyde adduct formation contributes to these pathogenic processes. Clearly, many key issues remain unresolved and warrant further investigation.

## Data Availability

No new data were created or analyzed in this study. Data sharing is not applicable to this article.

## Abbreviations

ADH	alcohol dehydrogenase
AFLD	alcoholic fatty liver disease
ALD	alcohol-associated liver disease
AMPK	AMP-activated protein kinase
BMI	body mass index
CYP2E1	cytochrome P450 2E1
DAMPs	damage-associated molecular patterns
DGAT1	diacylglycerol acyltransferase 1
DGAT2	diacylglycerol acyltransferase 2
ER	endoplasmic reticulum
FXR	farnesoid X Receptor
FLD	fatty liver disease
FFAs	free fatty acids
GST	glutathione S-transferase
HCC	hepatocellular carcinoma
HD	hepatogenic diabetes
HIF	hypoxia-inducible factors
IR	insulin resistance
LXR α	liver X receptor-α
MetALD	metabolic and alcohol -related fatty liver disease
MASLD	metabolic dysfunction-associated steatotic liver disease
MEOS	microsomal ethanol oxidizing system
NF-κB	Nuclear factor κB
PPRE	peroxisome proliferator–responsive element
PARPs	poly(ADP-ribose) polymerases
PPARα	peroxisome proliferator-activated receptor alpha
ROS	reactive oxygen species
SREBP1	Element-binding protein 1
TGs	Triglycerides
TNF- α	tumor necrosis factor-alpha
T2DM	type 2 diabetes mellitus
TNKS	tankyrases
WAT	visceral white adipose tissue
